# Baicalein Restores the Balance of Th17/Treg Cells via Aryl Hydrocarbon Receptor to Attenuate Colitis

**DOI:** 10.1155/2020/5918587

**Published:** 2020-10-05

**Authors:** Chang Liu, Yanyang Li, Yanping Chen, Shaowei Huang, Xiaojing Wang, Shuang Luo, Yulin Su, Lian Zhou, Xia Luo

**Affiliations:** School of Pharmaceutical Sciences, Guangzhou University of Chinese Medicine, China

## Abstract

As one of the ligands of aryl hydrocarbon receptor (AhR), baicalein, isolated from *Scutellaria baicalensis Georgi*, has been proved to exert potential therapeutic effects on ulcerative colitis (UC), but its therapeutic mechanism remains obscure. Authentically, ulcerative colitis can be alleviated by regulating the differentiation of naïve CD4^+^ T cells via AhR activation. So, our study planned to prove the hypothesis that baicalein protected mice against UC by regulating the balance of Th17/Treg cells via AhR activation. Immunofluorescence and western blot results showed that baicalein could promote AhR activation and induce it to transfer to the nucleus. We further determined the effect of baicalein on naïve CD4^+^ T cell differentiation *in vitro* by magnetic cell separation and drug intervention. The results showed that baicalein could promote Treg cell differentiation by activating AhR. *In vivo* study, UC mice were established by free drinking of dextran sulfate sodium (DSS) for 7 days and then were orally administrated by baicalein (10, 20, and 40 mg/kg), TCDD (AhR agonist), and CH223191 (antagonist). The results demonstrated that baicalein improved the symptoms of UC mice, regulated the balance of Th17/Treg cells, and restored the balance of proinflammatory cytokines such as IL-17, IL-6, and TNF-*α*; anti-inflammatory cytokines such as IL-10 and TGF-*β*; and epithelial protective cytokine IL-22 in UC mice, and these effects were related to AhR. Taken together, our research found that baicalein might be a potential drug for UC via regulating Treg cell differentiation and maintaining immune homeostasis and attempted to shed a light on the pivotal role of AhR in these effects.

## 1. Introduction

It is known that ulcerative colitis (UC) is a chronic nonspecific inflammatory disease, and its main diseased regions are the colon and rectum [1]. UC always seriously threatens the human health and social medical system due to its characteristics including various risk factors and recurrent attacks [2]. It is acknowledged that multiple factors cause antigens in intestinal luminal to cross the damaged epithelial barrier, which leads to the abnormal activation of the intestinal immune system and imbalance between anti-inflammatory and proinflammatory signals. Subsequently, a large number of leukocytes will migrate to the intestinal mucosa, thereby causing a sustained T cell immune response [3–5], which is common in UC. So, the current mainstream treatment concept is to affect the intestinal immune system, such as antieffector T cell activation or promoting anti-inflammatory signaling pathways [6].

The occurrence and development of UC are related to intestinal T cell dysfunction, mainly manifested as the imbalance of Th17/Treg cells [7]. Researchers found that the increase of Th17 cells in the lamina propria was accompanied by an altered structure of the intestinal crypts in UC patients [8]. Moreover, phenotypically and functionally changes in Treg cells as well as hyperfunction of Th17 cells were observed in UC patients [9]. Th17 cells, one of the subsets of T cells, are characterized by the secretion of the interleukin-17 (IL-17). IL-17 participates in the local inflammatory response by activating NF-*κ*B [10]. Besides, IL-17 will induce the production of inflammatory cytokines including IL-6, tumour necrosis factor-*α* (TNF-*α*), and chemokine such as CXCL8 [11], which will drive inflammatory cells to infiltrate tissue and lead to tissue damage. Consequently, it is generally believed that Th17 cells mainly mediate the proinflammatory response. CD4^+^ CD25^+^ Treg cells have multiple immune inhibitory functions, mainly reflecting in (1) expressing CD39 and CD73 to disrupt the metabolism of effector T cells, (2) secreting perforin and granzyme B to exert cytotoxic effects on effector T cells, and (3) promoting the secretion of anti-inflammatory cytokines such as IL-10 and TGF-*β*, thereby restraining the function of Th17 and Th1 cells [12]. CD4^+^ CD25^+^ Treg cells play a significant role in maintaining intestinal immune tolerance and alleviate inflammation [13], and a therapeutic strategy of using Tregs for controlling excessive immune response in UC has been proposed in recent years [14].

Many genes regulating Treg and Th17 cell differentiation are related to the pathogenesis of inflammatory bowel disease (IBD) [15], and aryl hydrocarbon receptor (AhR) is one of them. AhR, a ligand-dependent receptor, is an indispensable regulatory factor in immune cells and participates in the development of IBD. After AhR activation in intestinal epithelial cells (IECs) and intestinal immune cells, AhR will play a part in epithelial repair and inflammation inhibition. Therefore, AhR is considered as an important regulator of intestinal epithelial barrier and mucosal immunity [16, 17]. Previous studies clarified that AhR expression was downregulated in CD4^+^ in intestinal lamina propria [18] in group 3 innate lymphoid cells (ILC3s) in inflamed colon tissue of IBD patients [19]. A similar phenomenon was observed in DSS-induced colitis mice, while activation of AhR effectively alleviated colitis mice [20]. In animal studies, AhR^−/−^ mice were more susceptible to colitis induced by DSS than wild-type (WT) mice. Oral administration of AhR agonist *β*-naphthoflavone relieved inflammation and reduced proinflammatory cytokine levels (such as TNF-*α*, IL-6, and IL-1*β*) in the colon of WT mice [21]. Caspase recruitment domain family member 9 (CARD9) controls intestinal commensal bacteria that metabolize tryptophan into AhR agonist. Modified microbiota, reduced AhR activation, and IL-22 production were observed in CARD9^−/−^ mice, which lead to the impairment of intestinal immune homeostasis [22]. Such a series of evidence reflect that the AhR pathway has close relevance to IBD.

In addition, when AhR is specifically bound with exogenous ligands such as natural compounds including flavonoids and indoles, as well as endogenous ligands generated by host cells, microbiota, it is able to regulate Treg cell differentiation [23]. AhR agonist 2-(1′H-indole-3′-carbonyl)-thiazole-4-carboxylic acid methyl ester (ITE) activated AhR and induced the production of human Treg cells *in vitro*, as well as improved 2,4,6-trinitrobenzenesulfonic acid- (TNBS-) induced colitis humanized mice by upregulating the expression of CD39, granzyme B, and IL-10 *in vivo* [24]. AhR activated by 2,3,7,8-tetrachlorodibenzo-p-dioxin (TCDD) promoted the differentiation of naïve CD4^+^ T cell into Foxp3^+^ Treg cells, while inhibited Th1, Th2, and Th17 cell differentiation [25]. Therefore, inducing Treg cell differentiation via AhR activation will be an effective treatment strategy for IBD.

Baicalein, as a major active flavone derived from herbs *Scutellaria baicalensis Georgi*, has multiple effects such as anti-inflammatory and antibacterial [26]. Previous researchers found that baicalein dose dependently induced AhR expression in Hepa lclc7 cells, which was similar to AhR agonist [27]. Besides, baicalein has positive effects on intestinal inflammation [28]. Studies have shown that the effects of baicalein to activate AhR increased the expression of uridine 5′-diphosphate-glucuronosyltransferase 1A1 (UGT1A1) protein and its enzymatic activity in IECs and subsequently reduced intestinal inflammation [29]. In the intestinal damage murine model caused by ovalbumin (OVA) sensitization, baicalein increased the intestinal epithelial tight junction expression and maintained the function of intestinal epithelial barrier [30]. Therefore, we investigated the anticolitis efficacy of baicalein and explored the potential mechanisms that activating AhR could promote Treg cell differentiation and recover Th17/Treg balance in colitis mice.

## 2. Materials and Methods

### 2.1. Materials

Baicalein was purchased from Shanghai Macklin Biochemical Technology Co., Ltd. The purity of baicalein is 98% at least (≥98%). DSS was purchased from MP Biomedicals (California, USA). Dulbecco's Modified Eagle's Medium (DMEM) and fetal bovine serum (FBS) were purchased from Gibco (California, USA). AhR rabbit monoclonal antibody and cytochrome P450 1A1 (CYP1A1) rabbit monoclonal antibody were obtained from Cell Signaling Technology, Inc. (Boston, USA). The nuclear protein and cytoplasmic protein extraction kit and BCA protein assay kit were purchased from Beyotime Biotechnology (Shanghai, China). Mesalazine was purchased from the Guangdong Provincial Hospital of Chinese Medicine. CH223191 was purchased from APExBIO (Houston, USA). TCDD was purchased from Sigma-Aldrich (Missouri, USA). IL-10 and IL-17A enzyme-linked immune sorbent assay (ELISA) kits were purchased from MultiSciences Biotech (Hangzhou, China); IL-6, TNF-*α*, TGF-*β*, and IL-22 ELISA kits were purchased from Neobioscience Technology Co, Ltd. FITC-anti-CD4, APC-anti-CD25, PE-anti-Foxp3, and PEcy5.5-anti-IL-17A were purchased from eBioscience (San Diego, USA); CD3e and CD28 antimouse were purchased from BD Pharmingen (New Jersey, USA); mouse CD4^+^ CD62L^+^ T cell isolation kit and MACS-LS column were purchased from Miltenyi Biotec (Cologne, Germany). Foxp3/Transcription Factor Staining Buffer Set and Cell Stimulation Cocktail (plus protein transport inhibitors) (500x) was purchased from eBioscience (San Diego, USA). Recombinant murine IL-2 (rm IL-2), recombinant murine IL-6 (rm IL-6), and recombinant human TGF-*β*1 (rh TGF-*β*1) were purchased from PeproTech (New Jersey, USA). Mouse 1x Lymphocyte Separation Medium was purchased from Dakewe Biotech (Shenzhen, China).

### 2.2. Animals

Male C57BL/6 mice (aged 6-8 weeks) were purchased from Guangdong Medical Experimental Animal Center (Guangzhou, China). They were housed in a specific pathogen-free facility, in which the standard feeding condition included temperature (20 ± 2°C), humidity (55 ± 2%), and 12 h light/dark cycle. Mice got used to these conditions for at least 5 days before experiments. All experiments were conducted according to the guidelines approved by the Ethics Committee of Guangzhou University of Chinese Medicine.

### 2.3. DSS-Induced Colitis and Treatment

A total of 90 mice were randomly divided into 9 groups, 10 mice per group, including the control (sterile distilled water), model (sterile distilled water), mesalazine (600 mg/kg), baicalein (10, 20, and 40 mg/kg), TCDD (25 *μ*g/kg), CH223191 (10 mg/kg), and baicalein (40 mg/kg)+CH223191 (10 mg/kg) groups. All mice except for the control group were fed with 3% DSS for 7 days and sterile distilled water for the following 3 days, while mice in the control group were received with distilled water for 10 days. Baicalein and mesalazine were orally administered for 10 days; TCDD was intraperitoneally administered only on the first day; CH223191 was intraperitoneally administered for 10 consecutive days.

### 2.4. Assessment of Inflammation and Colitis Severity Disease Activity Index

The weight changes, water and food consumption, diarrhea, and hematochezia of mice were observed and recorded every day. The disease activity index (DAI) was abided by the scoring detailed regulations as follows: (a) body weight loss of 0%, 1-5%, 5-10%, 10-15%, and >15% was scored as 0, 1, 2, 3, and 4; (b) stool consistency: 0 = normal; 1 = loose stools; 2 = diarrhea; 3 = mild diarrhea; 4 = liquid stool; (c) hematochezia: 0 was scored for no blood in hemoccult (-); 1 for slightly positive hemoccult (+); 2 for positive hemoccult (++); 3 was scored for blood traces in stool visible (+++); 4 for gross rectal bleeding (++++) [31].

### 2.5. Histopathological Examination

On the 11^th^ day, all mice were euthanized. Their colons were collected and fixed in 4% formalin solution, dehydrated, embedded in paraffin, and sliced into 4 *μ*m slices. Then, the paraffin sections were stained with hematoxylin and eosin (H&E) for histological examination.

### 2.6. Immunofluorescence (IF) Analysis

EL-4 cells were seeded into 6-well plates at a density of 1 × 10^6^ cells/mL and divided into 6 groups including baicalein (25, 50 *μ*M), TCDD (5 nM), CH223191 (10 *μ*M), and CH223191 (10 *μ*M)+baicalein (50 *μ*M). After EL-4 cells were incubated with different drugs for 24 h, nuclear translocation of AhR was detected. The cells were fixed with 4% paraformaldehyde firstly. Then, the cells were washed with phosphate buffer saline (PBS) and permeabilized with 0.5% Triton X-100 for 10 min. Subsequently, they were blocked with 5% bovine serum albumin (BSA) for 30 min and incubated with an antibody against AhR at 4°C overnight. After being washed with PBS 3 times, cells were incubated with a goat anti-rabbit secondary antibody for 1 h. Finally, three times of washing were conducted, then the cells were stained with DAPI for 5 min, and images were obtained using a confocal laser scanning microscope (CLSM).

### 2.7. ELISA

On the 11^th^ day, the peripheral blood of mice was obtained and rested for 2 h, then centrifuged at 3000 rpm for 10 min. The supernatant was collected, and the levels of IL-6, TNF-*α*, IL-17, IL-10, TGF-*β*, and IL-22 were detected by using ELISA kits according to the manufacturer's instructions strictly.

### 2.8. Isolation of Colonic Lamina Propria Lymphocytes

The fresh colon tissue was cut into small pieces in a 10 mL centrifuge tube, and 5 mL digestion solution was added and shook gently at 37°C for 20 min to remove the epithelium and mucus. After aspirating supernatant gently, 3 mL collagenase IV solution (1 mg/mL) was added and gently shook at 37°C for 60 min. After digestion, the tissue suspension passed through 100 *μ*m and 70 *μ*m cell strainer in turn, and the filtrate was collected. Then, the filtrate was mixed with 3 mL PBS and centrifuged at 300g, 4°C for 10 min. The precipitations were resuspended with 3 mL of RPMI 1640 complete medium containing 1% penicillin-streptomycin solution and 5% fetal bovine serum. Then, the mixture was slowly added into the compounds, which contained 3 mL of 80% Percoll cell separation solution and 3 mL of 40% Percoll cell separation solution. After that, the mixture was centrifuged at 800g, 20°C for 20 min. The white separation interface was collected and mixed with 2 mL PBS, followed by centrifugation at 1000 rpm, 4°C for 10 min twice, and colonic lamina propria (LP) lymphocytes were obtained.

### 2.9. Flow Cytometry

On the 11^th^ day, mesenteric lymph nodes (MLNs) of mice were collected and mechanically ground into single-cell suspensions. After washing with cold PBS for 2 times, the cell concentration was adjusted to 10^7^ cells/mL and incubated with the cell stimulation cocktail (1 *μ*L/500 *μ*L) for 6 h. After that, the cells were incubated with FITC-conjugated anti-CD4 and APC-conjugated anti-CD25 at room temperature for 30 min. Then, the cells were fixed and permeabilized with the fixation/permeabilization working solution for 30 min. After washing with the permeabilization buffer, the cells were incubated with PEcy5.5-conjugated anti-IL-17A and PE-conjugated anti-Foxp3 for 30 min. Finally, the percentage of CD4^+^ IL-17^+^ cells and CD4^+^ CD25^+^ Foxp3^+^ cells were detected by using a flow cytometer.

### 2.10. Naïve CD4^+^ T Cell Separation

Naïve CD4^+^ T cells from the spleen of C57BL/6 mice were purified with magnetic beads (CD4^+^ CD62L^+^ T Cell Isolation Kit II). Briefly, C57BL/6 mice were euthanized, and the spleens were obtained under sterile condition, followed by manually grinding with 2 mL PBS into cell suspensions. Subsequently, mouse lymphocyte separation medium was used to separate lymphocytes from splenocytes. Then, the lymphocytes were washed with PBS and adjusted to 10^8^ cells/mL. 100 *μ*L of the biotin-antibody cocktail was added to cell suspension followed by incubation at 4°C for 10 min. Then, 300 *μ*L buffer combined with 200 *μ*L antibiotin microbeads was added and incubated at 4°C for 10 min. After that, the mixture was added into the MACS-LS column, and the filtrate denoting CD4^+^ T cells was collected. The filtrate was centrifuged and resuspended with 800 *μ*L buffer and mixed with 200 *μ*L CD62L microbeads. The mixture was incubated at 4°C for 10 min and then centrifuged and resuspended with 500 *μ*L buffer. Afterwards, the cell suspension was passed through the MACS-LS column and the filtrate was discarded. 1 mL buffer was flown into the column, and the filtrate denoting CD4^+^ CD62^+^ T cells was collected.

### 2.11. Detection of Cell Viability and Drug Toxicity

CD4^+^ T cells or EL-4 cells were seeded into 96-well plates at a density of 5 × 10^5^ cells/mL and treated with various concentrations of baicalein for 24 h. Then, 10 *μ*L 3-(4,5-dimethylthiazol-2-yl)-2,5-diphenyl-2H-tetrazol-3-ium bromide (MTT) solution (5 mg/mL dissolved in PBS) was added to the cells and incubated for 4 h. Finally, the supernatant was removed, and the precipitation was dissolved by 150 *μ*L DMSO. The absorbance was measured by an enzyme-labeled instrument.

### 2.12. Treg and Th17 Differentiation

Naïve CD4^+^ T cells were stimulated with anti-CD3e (10 *μ*g/mL), CD28 (2 *μ*g/mL), and rm IL-2 (50 IU) for 72 h. Under Th17 differentiation condition (rh TGF-*β*1 (5 ng/mL)), rm IL-6 (20 ng/mL) and CD4^+^ naïve T cells were, respectively, treated with baicalein (5 *μ*M and 10 *μ*M), TCDD (5 nM), CH223191 (10 *μ*M), and baicalein (10 *μ*M) combined with CH223191 (10 *μ*M) for 24 h. Under Treg differentiation condition (rh TGF-*β*1 (5 ng/mL)), CD4^+^ naïve T cells were, respectively, treated with drugs above. Afterwards, the cells were collected and stained with a fluorescent-conjugated flow antibody according to protocol 2.9. Finally, the frequencies of Th17 and Treg cells were detected by using the flow cytometry assay.

### 2.13. Western Blotting

The nuclear and cytoplasmic proteins in EL-4 cells were extracted, respectively, using the nuclear protein and cytoplasmic protein extraction kit. The nuclear and cytoplasmic protein concentration was detected, respectively, using a BCA protein assay kit. Then, the protein samples were separated by 10% separation gel and 5% concentrated gel electrophoresis at 80 V for 60 min. Afterwards, the proteins were then transferred onto a PVDF membrane at 300 mA for 2 h. The membrane was blocked with 5% skimmed milk for 2 h and then incubated with antibodies against AhR and CYP1A1 at 4°C overnight. After that, the membrane was washed with TBST three times and incubated with HRP-goat anti-rabbit secondary antibody at room temperature for 90 min, followed by three times of washing with TBST. Finally, the proteins on the membrane were detected by the chemiluminescence system.

### 2.14. Statistical Analysis

All data were analyzed by the GraphPad Prism 5.02 software. *P* values less than 0.05 (*P* < 0.05) were defined as a significant difference.

## 3. Results

### 3.1. Baicalein Activated AhR in EL-4 Cells

**Figure 1:**
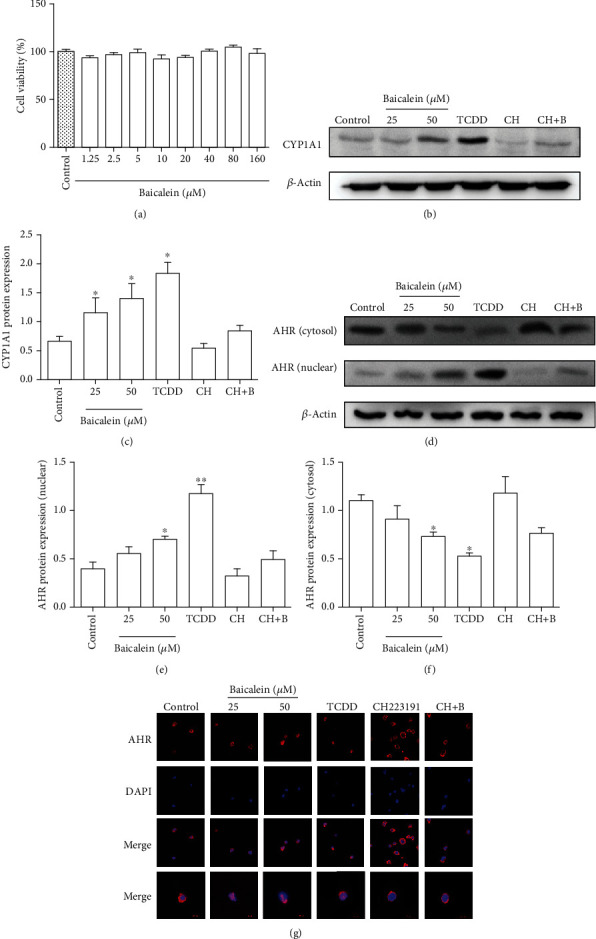
Baicalein activated AhR in EL-4 cells. (a) The viability and proliferation of EL-4 cells were detected by using MTT assays. (b–f) EL-4 cells were treated with baicalein (25 *μ*M and 50 *μ*M), TCDD (5 nM), CH223191 (10 *μ*M), and CH223191 (10 *μ*M)+baicalein (50 *μ*M) for 24 h; nuclear and cytoplasmic proteins of AhR and CYP1A1 were detected by using western blotting (WB), respectively. (g) The nuclear translocation of AhR was detected by using immunofluorescence (scale bar: 4 *μ*m). Experiments were repeated three times, and the results were expressed as means ± S.E.M.; ^∗^*P* < 0.05 and ^∗∗^*P* < 0.01 vs. control model. CH: CH223191; CH+B: CH223191+baicalein.

Firstly, we explored the activation of AhR by baicalein in EL-4 cells. Baicalein from 1.25 to 160 *μ*M showed little effect on EL-4 cell viability (Figure 1(a)). 50 *μ*M of baicalein significantly promoted the expression of CYP1A1 (AhR-upregulated modulator) (*P* < 0.05). The expression of AhR which was treated by 50 *μ*M of baicalein was increased in the EL-4 cell nucleus (*P* < 0.05), while decreased in the cell cytoplasm (*P* < 0.05), and this effect could be reversed by CH223191 (Figures 1(b)–1(f)).

In order to further observe the nuclear transport of baicalein on the AhR in EL-4 cells, IF was used. We found that AhR of the baicalein-treated EL-4 cells transferred from cytoplasm to the nucleus, which fails to be observed in the CH223191 and CH223191+baicalein groups (Figure 1(g)). All these results indicated that baicalein bound to AhR induced it to transfer to the cell nucleus and promoted the expression of downstream target gene CYP1A1.

### 3.2. Baicalein Alleviated Symptoms of Ulcerative Colitis Mice Induced by DSS

**Figure 2:**
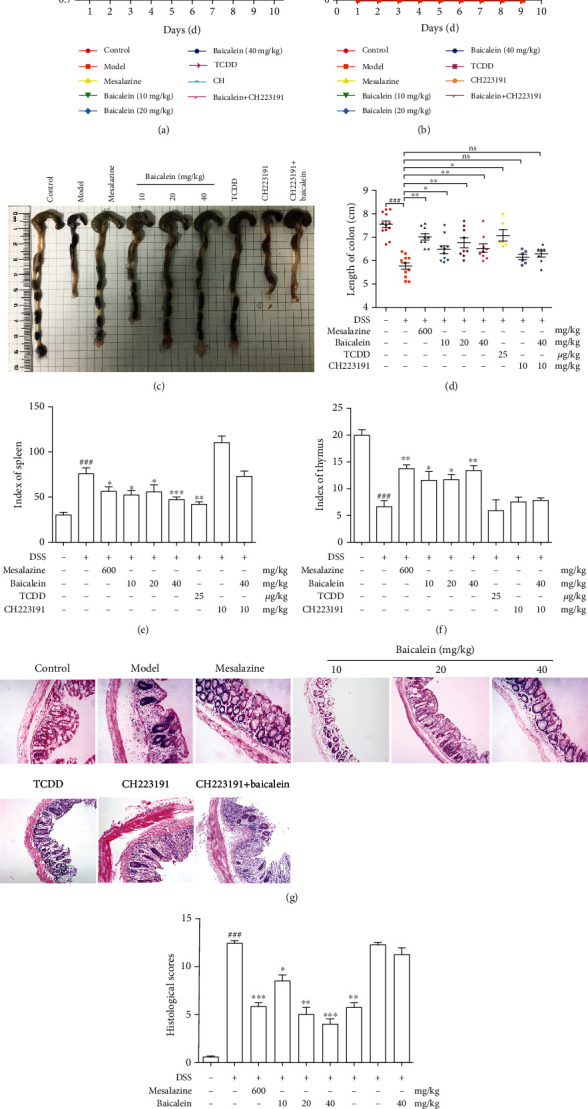
Baicalein ameliorated disease activity in mice with DSS-induced colitis. Mice were orally administrated of 3% DSS for seven days and followed by sterile distilled water alone for another three days. Baicalein (10, 20, and 40 mg/kg) and mesalazine (600 mg/kg) were orally administered daily for 10 consecutive days. Mice were treated by oral administration of baicalein (40 mg/kg) and intraperitoneal injection of CH223191 (10 mg/kg) for 10 consecutive days and intraperitoneal injection of TCDD (25 *μ*g/kg) on day 1. On the 11^th^ day, mice were sacrificed, and colons were collected. (a) Bodyweight changes were evaluated in each group. (b) Disease activity index (DAI) scores were evaluated in each group. (c, d) The colon lengths were evaluated in each group. (e, f) The spleen and thymus index were calculated. #*P* < 0.05, *^##^P* < 0.01, and *^###^P* < 0.001 vs. the control group. ^∗^*P* < 0.05, ^∗∗^*P* < 0.01, and ^∗∗∗^*P* < 0.001 vs. the model group. (g, h) The histological changes were detected by using H&E staining, and histological activity index (HAI) was evaluated in each group (100x original magnification). Results were expressed as means ± S.E.M.#*P* < 0.05, *^##^P* < 0.01, and *^###^P* < 0.001 vs. the control group. ^∗^*P* < 0.05, ^∗∗^*P* < 0.01, and ^∗∗∗^*P* < 0.001 vs. the model group.

As expected, loss of weight was observed in mice that were received with DSS. On the last day, compared with mice in the control group, mice in the model group had the most significant weight loss (*P* < 0.001) and dramatic increased DAI scores. Compared with the model group, the weight loss of the mice in the baicalein (10, 20, and 40 mg/kg) group was slowed down (*P* < 0.05, *P* < 0.05, and *P* < 0.001) and DAI scores of them were decreased (*P* < 0.05, *P* < 0.05, and *P* < 0.001; Figures 2(a) and 2(b)). Furthermore, 3% DSS significantly shortened the colons of mice (*P* < 0.001) and baicalein could prolong them (Figures 2(c) and 2(d)). The spleen index of mice in the model group significantly rose, and the thymus index significantly lowered (*P* < 0.001 and *P* < 0.01). Compared with the model group, baicalein had a significant recovery effect on the spleen index and thymus index (*P* < 0.01and *P* < 0.001, Figures 2(e) and 2(f)). H&E staining in colitis murine colons further revealed obvious pathological changes, including severe damage in the surface epithelium, disappearance of crypt structure, and infiltration of inflammatory cells, and histopathological scores of colitis mice were dramatically higher than mice in the control group (*P* < 0.001). Baicalein displayed significant improvement in murine colonic histological structure (*P* < 0.05, *P* < 0.01, and *P* < 0.001; Figures 2(g) and 2(h)).

### 3.3. Baicalein Restored the Th17/Treg Balance in Colitis Mice

**Figure 3:**
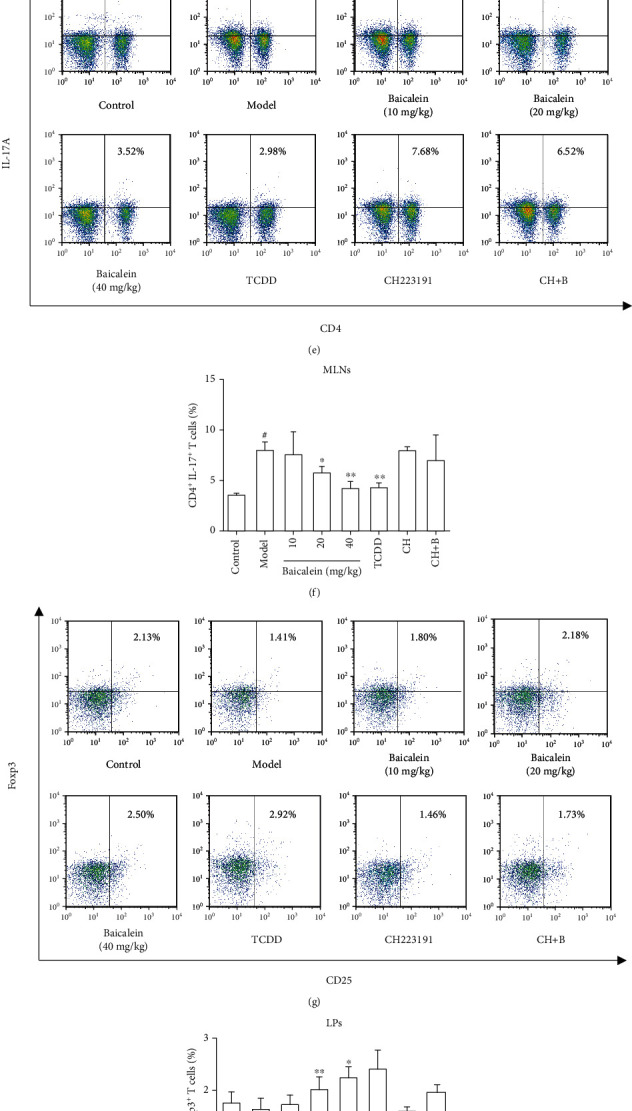
Effect of baicalein on the imbalance of Th17/Treg of UC mice. (a, b) CYP1A1 protein expression of the colon in UC mice was detected by WB. (c–f) The percentages of CD4^+^ IL-17^+^ cells in MLNs and LPs were detected by flow cytometry. (g–j) The percentages of CD4^+^ CD25^+^ Foxp3^+^ cells in MLNs and LPs were detected by flow cytometry.

Firstly, we investigated whether oral administration of baicalein promoted the AhR activation in the colon of UC mice. WB results showed that in the UC state, CYP1A1 protein expression was slightly downregulated. 20 mg/kg and 40 mg/kg of baicalein obviously upregulated CYP1A1 protein expression in UC mice (*P* < 0.05, Figures 3(a) and 3(b)). The imbalance of Th17/Treg is a critical pathologic change during the occurrence and development of UC, so flow cytometry was used to detect the proportion of Th17 and Treg cells in the LP and MLNs of UC mice. In LP, compared with the control group, the proportions of Th17 cells in the model group were increased, and baicalein could reduce them in varying degrees, especially 40 mg/kg of baicalein had a significant effect (*P* < 0.05). And compared with the control group, the proportions of Treg cells in the model group decreased slightly and increased after oral administration of baicalein, 20 and 40 mg/kg of which had statistical difference (*P* < 0.05, *P* < 0.01). Similar results were obtained from the proportions of Treg cells and Th17 cells in MLNs (Figures 3(c)–3(j)).

### 3.4. Baicalein Restored Cytokines in the Serum of DSS-Induced Colitis Mice

**Figure 4:**
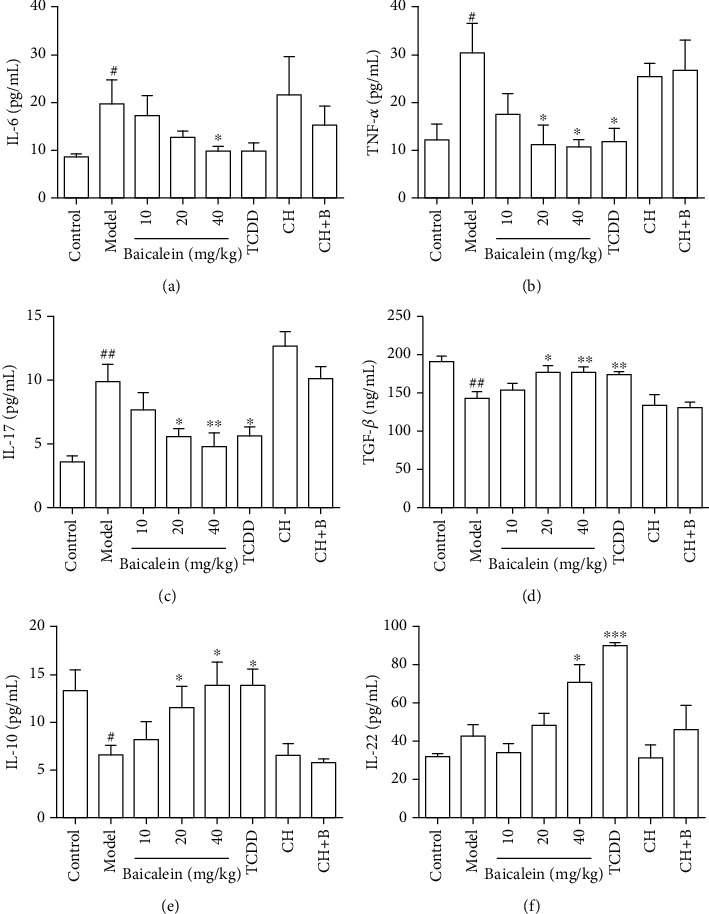
Effects of baicalein on cytokines in the serum of DSS-induced colitis mice: (a) IL-6; (b) TNF-*α*; (c) IL-17; (d) TGF-*β*; (e) IL-10; (f) IL-22. Results were means ± S.E.M.#*P* < 0.05 and *^##^P* < 0.01 vs. control group; ^∗^*P* < 0.05, ^∗∗^*P* < 0.01, and ^∗∗∗^*P* < 0.001 vs. model group. CH: CH223191; CH+B: CH223191+baicalein.

In order to further observe the anti-inflammatory effect of baicalein, we detected the content of proinflammatory cytokines TNF-*α*, IL-6, and IL-17; anti-inflammatory cytokines IL-10 and TGF-*β*; and epithelial protective cytokine IL-22 in the serum of mice in each group. Compared with the control group, the levels of TNF-*α*, IL-6, and IL-17 were found increased while the levels of IL-10 and TGF-*β* were found decreased markedly in the model group. Baicalein (20 and 40 mg/kg) obviously downregulated levels of TNF-*α*, IL-6, and IL-17 (Figures 4(a)–4(c)). Meanwhile, baicalein (40 mg/kg) significantly upregulated levels of IL-10, TGF-*β*, and IL-22 in the serum of colitis mice (*P* < 0.05, *P* < 0.01, and *P* < 0.05; Figures 4(d)–4(f)). The results above demonstrated that baicalein could effectively mitigate the inflammatory state in colitis mice.

### 3.5. Baicalein Could Directly Regulate the Differentiation of Treg In Vitro

**Figure 5:**
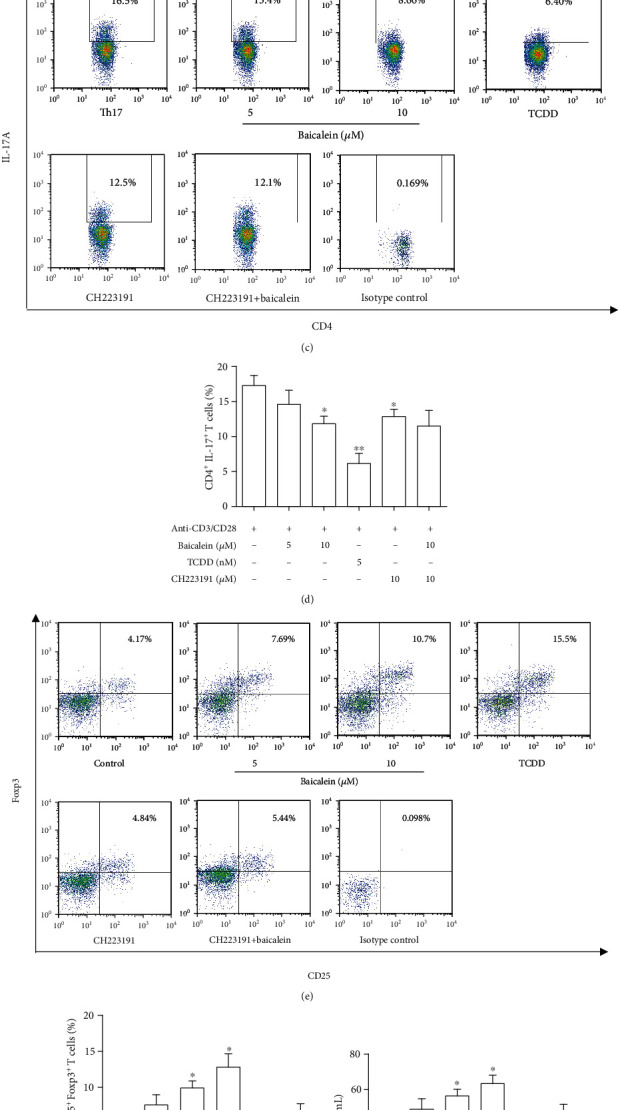
Effect of baicalein on the differentiation of Th17 and Treg cells *in vitro*. Naïve CD4^+^ T cells were treated with anti-CD3/CD28 (2 *μ*g/mL) and baicalein (5 and 10 *μ*M), TCDD (5 nM), and CH223191 (10 *μ*M) under Treg or Th17 cell differentiation condition for 24 h; percentages of CD4^+^ IL-17^+^ cells CD4^+^ CD25^+^ Foxp3^+^ cells were detected by flow cytometry. (a) Naïve CD4^+^ T cells were separated and identified by flow cytometry. (b) The viability and proliferation of naïve CD4^+^ T cells were detected by using MTT assays. (c, d) The percentages of CD4^+^ IL-17^+^ cells were detected by using flow cytometry. (e, f) The percentages of CD4^+^ CD25^+^ Foxp3^+^ cells were detected by flow cytometry. (g, h) The levels of IL-17 and IL-10 in the supernatant were detected by using ELISA. Experiments were repeated three times, and results were expressed as means ± S.E.M.^∗^*P* < 0.05 and ^∗∗^*P* < 0.01 vs. control model. CH: CH223191; CH+B: CH223191+baicalein.

To further confirm the above-mentioned results, naïve CD4^+^ T cells in the spleen were isolated, identified, and induced differentiation. The identification results indicated that the purity of naïve CD4^+^ T cells isolated was up to 95.2% (Figure 5(a)). To prevent inhibition of baicalein on cell viability and proliferation, an MTT experiment was performed. At concentrations of 1.25, 2.5, 5, and 10 *μ*M, baicalein showed little effects on naïve CD4^+^ T cell viability. So, we selected 5 and 10 *μ*M of baicalein for the following experiments (Figure 5(b)). Under Th17 cell differentiation conditions, Th17 cell differentiation was repressed after treatment of baicalein, and 10 *μ*M of baicalein had an obvious effect (*P* < 0.05). Under Treg cell differentiation conditions, 10 *μ*M of baicalein significantly promoted the differentiation of naïve CD4^+^ T cells into Treg cells. CH223191 had no effects on Treg cell differentiation, and it could reverse the effect of baicalein. Interestingly, CH223191 could inhibit Th17 cell differentiation (*P* < 0.05), which showed that AhR participated in Th17 cell differentiation (Figures 5(c)–5(f)). Naïve CD4^+^ T cell differentiation comes with the secretion of cytokines, so we detected the levels of IL-10 and IL-17A during the cell differentiation. Compared with the control group, 10 *μ*M of baicalein-treated naïve CD4^+^ T cell secreted lots of IL-10 under Treg cell differentiation conditions (*P* < 0.05), while it was able to inhibit IL-17A secretion during Th17 cell differentiation (*P* < 0.05). Baicalein-induced Treg differentiation can be inhibited by CH223191, indicating that baicalein induced Treg cell differentiation by activating AhR (Figures 5(g) and 5(h)).

### 3.6. Baicalein-Mediated Alleviation of Colitis Mice Is an AhR-Dependent Manner

We used AhR agonist TCDD, CH223191 to verify that the effects of baicalein on the treatment of colitis mice are an AhR-dependent manner. We observed that TCDD had positive effects on clinical symptoms including weight loss, DAI scores, colon length, colon pathological changes, and Th17/Treg cell balance in LPs and MLNs, while CH223191 had negative effects and offset the anticolitis effects of baicalein. These results suggested that baicalein alleviated colitis mice via AhR activation (Figures 2–4).

## 4. Discussion

Ulcerative colitis is identified as an etiologically complicated inflammatory disease. During the disease course, intestinal immune homeostasis is damaged, including the imbalance between anti-inflammatory and proinflammatory signals, which is manifesting as the overactive response of Th17 cells and the insufficient response of Treg cells. This persistent imbalance further aggravates UC symptoms through the increased secretion of inflammatory cytokines and decreased secretion of anti-inflammatory cytokines, which is reflected in our experimental results. Therefore, restoring the balance of anti-inflammatory and proinflammatory signals and remodelling the intestinal immune homeostasis can be used for the treatment of UC [14]. So, current clinical therapies focus on reducing excessive inflammation and regulating immune homeostasis. In a normal physiological state, activated lymphocytes leave the intestine through regional lymph nodes and reenter the intestine via specific homing mechanisms [6]. When UC occurs, a large number of naïve CD4^+^ T cells, effector T cells, and memory T cells will migrate to the intestine and then accumulate and expand locally [32, 33]. Therefore, the proportion of Th17/Treg cells in MLN and LP and related cytokines detected to reflect the inflammatory state in the colon of colitis mice.

We found that baicalein had effective effects on relieving colitis mice, including significantly lowering the DAI scores alleviating the tendency of weight loss, restoring the spleen and thymus index, protecting the integrity of colonic histological structure, and reducing inflammatory cell infiltration. In addition, we also found that baicalein played an important role in regulating Th17/Treg cell balance; increasing IL-10, TGF-*β*, and IL-22; and reducing TNF-*α*, IL-6, and IL-17. IL-6 recruits various immune cells; promotes the differentiation and survival of Th1, Th2, or Th17 cells; and induces them to produce cytokines, which amplify intestinal inflammation [34]. TNF-*α* acts on the intestinal wall, alters the tight junction function, induces apoptosis of IECs [35], and regulates T cell apoptosis [36]. IL-10 prevents damage to the host by limiting the immune cell response to pathogens [37]. IL-22, a kind of important protection factor, maintains IEC survival, proliferation, and program production of antimicrobial peptides, which keeps the integrity of mucosa barrier [38]. It was worth noting that baicalein increased the production of IL-22, which might be related to AhR activation in ILC3 [18, 39]. Therefore, we speculated that baicalein could exert an immunosuppressive effect by regulating Th17/Treg cell balance and cytokine levels in the intestinal immune microenvironment.

Naïve CD4^+^ T cells, a kind of precursor cell derived from T helper cells and regulatory T cells, are capable of differentiating into Treg and Th17 cells under the stimulation of IL-6 and TGF-*β* stimulation. These cells mainly secrete proinflammatory cytokine IL-17 and anti-inflammatory cytokine IL-10, respectively, which are involved in normal physiological and inflammatory states. The imbalance of Th17/Treg cells in UC may be on account of the fact that a variety of signals (including cytokines, metabolites, and microbial) *in vivo* are disordered, causing naïve CD4^+^ T cells to differentiate into Th17 cells and producing more proinflammatory cytokines. Therefore, we planned to inhibit the differentiation of Th17 cells and promote the differentiation of Treg cells through drug intervention to achieve the purpose of relieving UC. Interestingly, our data showed that baicalein intervention could promote Treg cell differentiation and inhibit Th17 cell differentiation *in vitro*.

AhR is considered as a key regulator of intestinal immune response, and it is crucial to intestinal immune homeostasis. When AhR ligands are specifically bound to it, the AhR complex transfers to the nucleus and then recognizes the promoters containing specific enhancer sequences known as xenobiotic response elements (XREs), thereby controlling downstream genes such as CYP1A1, CYP1A2, and CYP1B1. Immunofluorescence results suggested that baicalein interference induced AhR to translocate to the nucleus. WB results showed that baicalein increased AhR downstream protein expression of the CYP1A1 level and increased the expression of AhR in the nucleus in a concentration-dependent manner. These results were consistent with previous researches [27] and indicated that baicalein may be a potential ligand for AhR.

On the one hand, AhR participates in immune regulation by controlling Treg cell differentiation and their activities. AhR agonist TCDD induced demethylation of CpG islands located on the Foxp3 promoter, promoted the expression of Foxp3, and induced Treg cell differentiation [40]. However, AhR inhibitor CH223191 restrained Treg cell differentiation [24, 41], suggesting that AhR directly affects Treg cell differentiation. Therefore, we speculated that baicalein induced Treg cell differentiation via activating AhR *in vitro*. As expected, our *in vitro* study showed that AhR activation induced by baicalein promoted naïve T cells to differentiate into Treg cells, and this effect was reversed by CH223191. On the other hand, AhR also takes part in Th17 cell differentiation. Previous research showed that AhR activation by 6-formylindolo[3,2-b] carbazole (FICZ) boosted Th17 cell differentiation and aggravated encephalomyelitis [25]. In addition, AhR is able to upregulate the Aiolos gene to weaken the differentiation inhibition of IL-2 on Th17 cell [42]. Our results showed that CH223191 inhibited Th17 cell differentiation, which is consistent with previous studies [43], explaining that AhR is necessary for Th17 cell differentiation and baicalein-induced Th17 cell differentiation inhibition may be directly related to other mechanisms. AhR selectively combines STAT1 and STAT5 to negatively regulate Th17 cell differentiation under Th17 cell differentiation condition (TGF-*β*^+^ IL-6 or TGF-*β*^+^ IL-21) [44]. We found that baicalein inhibited Th17 cell differentiation, and the underlying mechanism might be affecting the expression of STAT1 or STAT5, but further studies are needed.

**Figure 6:**
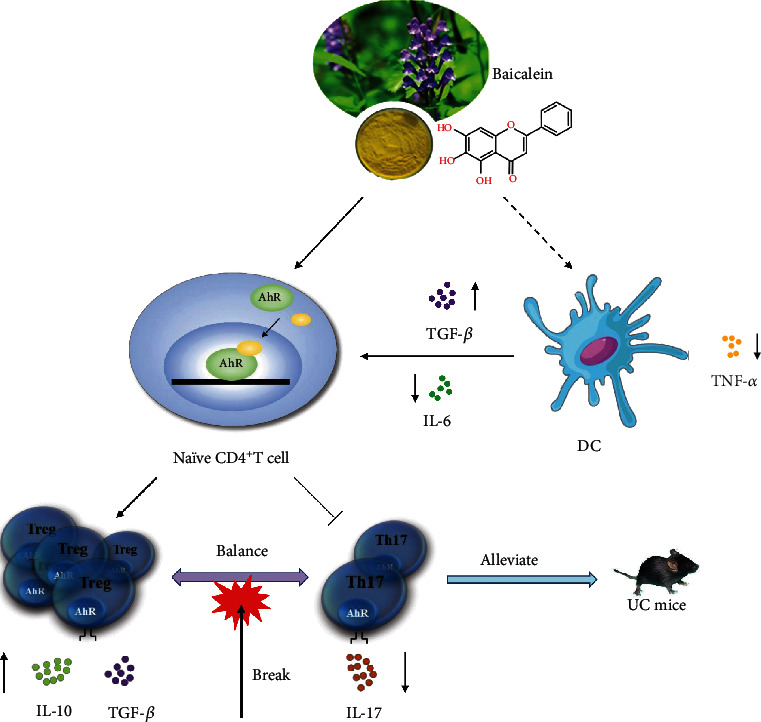
Schematic representation of the mechanism in baicalein-induced Treg cell differentiation through AhR against UC. The balance of Th17/Treg cells in the intestinal of DSS-induced colitis mice was disrupted, leading to hyperactive proinflammatory response, insufficient anti-inflammatory response, aggravating intestinal inflammation, and tissue damage. Baicalein promoted Treg cell differentiation by activating AhR. Meanwhile, baicalein reduced levels of IL-6 and increased levels of TGF-*β* in colitis mice, which impeded the initiation of Th17 cell differentiation. Moreover, baicalein regulated the balance of proinflammatory cytokines such as IL-17, IL-6, and TNF-*α* and anti-inflammatory cytokines such as IL-10 and TGF-*β*, which controlled intestinal immune homeostasis and reduced inflammatory damage.

AhR interacts with Foxp3 to strengthen the binding of AhR DNA at the orphan chemoattractant receptor Gpr15 locus and enhance the expression of Gpr15, thereby promoting Treg cells homing to the large intestine, which is important for intestinal immune homeostasis [45]. It is one reason why AhR expression in intestinal Treg cells is much higher than that of Treg cells in other anatomical sites [46]. It is acknowledged that exogenous agonist TCDD inhibits excessive immune response [47], which is associated with the induction of Treg cell differentiation [48, 49]. In order to demonstrate that the effects of protective roles that baicalein played in the treatment of UC are an AhR-dependent manner, we used CH223191 combined with baicalein to prove our speculation *in vivo*. We noticed that CH223191 had little effects on anticolitis and regulating the Th17/Treg cell balance and it was capable to reverse the positive effects of baicalein. Therefore, we speculated that baicalein induced naïve CD4^+^ T cells to differentiate into Treg cells in the colon via AhR activation. Dendritic cells (DCs), one of two major types of antigen-presenting cells (APCs), respond to antigens by producing IL-6 and TGF-*β*, which initiate the differentiation of naïve T cells to Th17 cells. However, reducing stimulation of IL-6 represses Th17 cell differentiation [50] and increasing stimulation of TGF-*β* promotes Treg cell differentiation [51]. We observed that baicalein developed the secretion of TGF-*β* and suppressed the production of IL-6 in colitis mice. We considered that baicalein might affect the capability of DCs to produce IL-6 via AhR because AhR activation in DCs restrains the production of IL-6 [52–54]. But the specific relevance between baicalein and DCs during cytokine production should be further investigated. Furthermore, baicalein inhibited Th17 cell differentiation by affecting its differentiation environment including reduction of IL-6 and increase of TGF-*β* levels. Under the common influence of these two aspects, baicalein regulated Th17/Treg cell balance, restored the balance of proinflammatory and anti-inflammatory cytokines, exerted immunosuppressive effects, and regulated immune homeostasis (Figure 6).

## 5. Conclusions

In conclusion, our study suggested that baicalein could protect mice from DSS-induced colitis, and its therapeutic mechanism may be related to the regulation of Th17/Treg differentiation via AhR activation.

## Data Availability

The data used and analyzed in this paper can be obtained from the corresponding authors with reasonable requests.
